# Increasing incidence of breast cancer in young women over time

**DOI:** 10.1016/j.breast.2025.104555

**Published:** 2025-08-05

**Authors:** Pascal Pujol, Laurent Remontet, Bénédicte Lapôtre-Ledoux, Agnès Rogel, Lionel Lafay, Florence Molinié

**Affiliations:** a Department of Cancer Genetics, University of Montpellier, Montpellier, France; b SFMPP, Montpellier, France; c Service Biostatistiques-bioinformatique, Hospices Civils de Lyon, Lyon, France; d Registre Du Cancer de La Somme, Service Épidémiologie Hygiène et Santé Publique, CHU Nord, Amiens, France; e Francim, French Network of Cancer Registries, Toulouse, France; f Santé Publique France, Saint-Maurice, F-94415, France; g Direction de L'observation des Sciences des Données et de L'évaluation, Institut National Du Cancer, Boulogne-Billancourt, France; h Loire-Atlantique/Vendée Cancer Registry, CHU Nantes, Nantes, France; i CERPOP, Université de Toulouse, Inserm, UPS, Toulouse, France

**Keywords:** Breast cancer, Young women, Incidence

## Abstract

The incidence of early-onset breast cancer (EOBC) has recently been shown to be increasing over time in the US and the UK.

Using national cancer registries data including 229,352 BC cases, we show that the incidence rate of EOBC in France increased steadily from 1990 to 2023, rising from 16.1 (95 % CI: 14.7–17.8) to 26.3 (95 % CI: 20.7–33.3) and from 98.7 (95 % CI: 93.8–103.7) to 131.2 (95 % CI: 115.8–148.7) per 100,000 person-years in women aged 30 and 40 years, respectively.

This population-based study confirms that the incidence of EOBC is increasing over time in Western countries. Further research is needed to explain this trend, which may have implications for prevention and screening strategies.

## Highlights

• Incidence of early onset breast cancer is increasing over time in Western countries.
• Screening guidelines may take into account the rising incidence of breast cancer in young women.
• Changes in hormonal factors, lifestyle, food habits, genetics or unknown factors may be involved.

## 1 Introduction

Recent publications have shown that the incidence of early-onset breast cancer (EOBC) has been increasing in the US and UK over the past decade [[1], [2], [3]]. It has also been shown that the overall incidence of breast cancer in Europe has been rising over the same period of time [4]. This increased incidence, particularly in young women, may reflect important changes in risk factor exposure and lifestyle over time and is a current public and media concern.

In France, cancer surveillance is organized through a partnership agreement between the French Network of Cancer Registries (Francim), the French Agency for Public Health, the French National Cancer Institute (INCa), and the Biostatistics and Bioinformatics Unit of the Hospices Civils de Lyon (HCL). The most recent study describing French national estimates of cancer incidence trends focused on the period 1990–2023 [5]. Here, we present data on breast cancer incidence by age from the most recent national study mentioned below.

## 2 Methods

Observed incidence data between 1985 and 2018 were obtained from the French cancer registries of the Francim network [6]. The quality and completeness of these population-based registries are certified every five years with an audit by the National Evaluation Committee of Registries [7]. This cohort is composed of individuals from the twenty district registries available in France. An average of 5,280,427 persons were assessed per year during the follow-up period. A total of 229,352 BC cases were observed during this period.

National incidence was estimated from a Poisson mixed-effects model including the effects of age and year of diagnosis using penalized splines, and a random department effect [8]. Incidence was estimated from 1990 to 2018 and projected from 2019 to 2023, excluding the potential impact of the Covid-19 pandemic. Age-specific incidence rates were expressed as the number of cases per 100,000 person-years (PY), and the rate was age-standardized for the whole population (age-standardized rate: ASR), using the age structure of the world population as a reference [9]. Trends in age-specific rates (for six given age at diagnosis: 30, 40, 50, 60, 70 and 80 years old) and ASR were summarized by their average annual percentage change (AAPC) over the period 1990–2023. Further methodological details can be found in a previous article [5].

## 3 Results

**Fig. 1:**
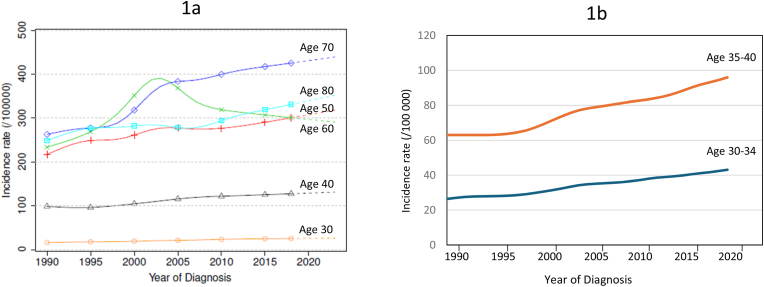
Trends in female breast cancer incidence in France between 1990 and 2023 by age at diagnosis.

Analysis of French cancer registry data shows that the national estimated incidence rate of EOBC has increased steadily from 1990 to 2023: from 16.1 (95 % CI: 14.7–17.8) to 26.3 (95 % CI: 20.7–33.3) per 100,000 person-years (PY) for women aged 30 years and from 98.7 (95 % CI: 93.8–103.7) to 131.2 (95 % CI: 115.8–148.7) PY for women aged 40 years (Fig. 1a). This corresponds to a mean annual percentage change of 1.5 % (95 % CI: 0.7–2.3) and 0.9 % (95 % CI: 0.5–1.3), respectively. As shown in Table 1, a similar trend of steady increase was also observed for the total population (ASR from 72.8 in 1990 (95 % CI: 70.9–74.8) to 99.2 (95 % CI: 95.6–102.9) in 2023) and for most other age groups (50, 70, 80), except for the group of 60 years. Analysis by five-year age group showed similar trends, with steadily increasing BC rates in the 30–34 and 35–40 age groups (Fig. 1b).

**Table 1:** Age-specific incidence rate and ASR per 100,000 PY between 1990 and 2023 and their average annual percentage change (AAPC).

Age	Incidence rate^a^ 1990	Incidence rate^a^ 2023	AAPC∗(%) 1990–2023	(CI95 %)
**All age**	72.8 (70.9; 74.8)	99.2 (95.6; 102.9)	**0.9**	(0.8; 1.0)
**30**	16.1 (14.7; 17.8)	26.3 (20.7; 33.3)	**1.5**	(0.7; 2.3)
**40**	98.7 (93.8; 103.7)	131.2 (115.8; 148.7)	**0.9**	(0.5; 1.3)
**50**	217.2 (207.9; 226.9)	318.6 (291.9; 347.7)	**1.2**	(0.9; 1.4)
**60**	233.9 (224.4; 243.9)	291.1 (267.3; 317)	**0.7**	(0.4; 0.9)
**70**	263.1 (251.5; 275.2)	439.3 (404.6; 476.9)	**1.6**	(1.3; 1.8)
**80**	248.9 (236.9; 261.6)	352.1 (316.6; 391.5)	**1.1**	(0.7; 1.4)

a ASR for “All Age” line.

## 4 Discussion

Our results confirm an increasing incidence of EOBC in France over time, as observed in the USA and the UK [[1], [2], [3],^5^]. According to the American Cancer Society report, the average annual percentage change in breast cancer incidence has steadily increased by 1.4 % from 2012 to 2021 [10]. In the most recent decade (2012–2021), the increase in incidence among younger women was faster than among older women (1.4 % vs. 0.7 % per year) [1]. In the United Kingdom, cancer incidence among people aged 25–49 years increased by 22 % between 1993-95 and 2016-18, while incidence among people aged 75 years and older increased by 9 % [3]. A global increase in the incidence of BC cancer is also observed in northern Europe according to the NORDCAN data base, compiling of cancer registries from Denmark, Finland, Norway and Sweden [11].

Since young women are not usually screened for breast cancer, this raises the question of increased exposure of young girls or women to factors affecting the risk of premenopausal breast cancer over the last decades. The main known determinants of breast cancer related to hormonal factors followed parallel trends such as increasing age at first childbirth, younger age at first period, decreasing fertility rate, decreasing breast feeding and rising use of oral contraception [12,13]. For instance, the fertility rate declined from 69.4 births per 1000 women in 2007 to an all-time low of 54.4 births per 1000 women in 2023 [13]. There has also been a shift toward later age at first birth, which is associated with an increased risk of HR-positive breast cancer [14]. In the US population, the rising trend of BC is mostly confined to HR-positive disease [1]. We also previously reported on a rising level of estrogen receptor positivity of breast tumors over time, which could reflect these changes at the biological level [15]. One limitation of our study is that data on hormone receptor expression was not available to analyse the hormone status of early breast cancer over time. However, in the population-based cross-sectional study by Xu et al. [2], the increasing breast cancer incidence rates in the US among women aged 20–49 years, was mainly due to increases in the incidence of ER + tumors, as incidence of ER− tumors decreased [2].

Changes in hormonal factors may not fully explain the increasing incidence of EOBC. Another possible explanation for the continuous increase in the incidence of BC and EOBC may be the remarkable changes in lifestyle that have taken place in recent decades. These changes may include dietary habits, alcohol consumption, exposure to radiation and pollutants, stressful and urban lifestyles, sedentary lifestyles, or unknown factors. Finally, genetic or epigenetic factors could not be excluded, such as an increase in *de novo* germline mutations due to male and reproductive aging, or an increase in somatic mutations or epigenetic events under environmental stress.

Because of the increase in younger-onset breast cancer, the US Preventive Services Task Force and the American Cancer Society lowered the recommended age to begin biennial mammography screening from 50 to 40 years in 2024 [10,16]. This is not the case in Europe, where the ECIBC's Guidelines Development Group (GDG) recommends against mammography screening for asymptomatic women aged 40–44 years at average risk for breast cancer [17], because the undesirable health effects of screening are large and the desirable effects are small. However, European recommendations have lowered the age for the first invitation to organized breast cancer screening from 50 to 45 years old. France has not yet changed its policy of inviting women for biannual mammography screening for women aged 50 to 74.

Further research is needed to investigate this increase in incidence, which may have implications for knowledge of breast risk factors, screening and prevention.

## CRediT authorship contribution statement

**Pascal Pujol:** Writing – original draft, Supervision, Conceptualization. **Laurent Remontet:** Writing – review & editing, Methodology, Formal analysis. **Bénédicte Lapôtre-Ledoux:** Resources, Data curation. **Agnès Rogel:** Resources. **Lionel Lafay:** Resources. **Florence Molinié:** Writing – review & editing, Resources, Methodology.

## Declartion of competing interest

Declaration of Competing Interest Pascal Pujol: Honoraria for lectures, presentations, speakers bureaus, manuscript writing or educational events from AstraZeneca, Pfizer, Guardant, MSD, Novartis, Johnson et Johnson, and Seqone. Consulting fees from Exact Sciences; Laurent Remontet, Bénédicte Lapôtre-Ledoux, Agnès Rogel, Lionel Lafay, Florence Molinié declare no competing interests.
